# Bis[μ-3,5-bis­(2-pyrid­yl)pyrazolato]bis­(hydrogensulfato)­dicopper(II) methanol disolvate

**DOI:** 10.1107/S1600536811038700

**Published:** 2011-10-12

**Authors:** Akio Mishima, Akira Fuyuhiro, Hitoshi Kumagai, Satoshi Kawata

**Affiliations:** a Department of Chemistry, Faculty of Science, Fukuoka University, Nanakuma, Jonan-ku, Fukuoka 814-0180, Japan; b Department of Chemistry, Graduate School of Science, Osaka University, Toyonaka, Osaka 560-0043, Japan; c Institute for Molecular Science, 38 Nishigounaka Okazaki, Aichi 444-8585, Japan

## Abstract

The title compound, [Cu_2_(C_13_H_9_N_4_)_2_(HSO_4_)_2_]·2CH_3_OH, consists of discrete centrosymmetric dinuclear complex mol­ecules and methanol solvent mol­ecules. The Cu^II^ atom shows a square-pyramidal coordination geometry and is bonded to four N atoms of the two bis-chelating 3,5-bis­(2-pyrid­yl)pyrazol­ate ions (bpypz^−^) and one O atom of the hydrogensulfate ion. The bpypz^−^ ligands in the complex mol­ecule are virtually coplanar [dihedral angle between the mean ligand planes =  0.000(1)°] with the Cu^II^ atom deviating in opposite directions from their best plane by 0.2080 (12) Å. π–π stacking inter­actions between the pyridyl and pyrazole rings [centroid–centroid distance = 3.391 (3) Å] and strong O—H⋯O hydrogen bonds between the hydrogensulfate ligands and the methanol mol­ecules assemble the mol­ecules into a one-dimensional polymeric structure extending along the *a* axis. The methanol mol­ecule acts both as an accepter and a donor in the hydrogen bonding.

## Related literature

For metal complexes of 3,5-bis­(2-pyrid­yl)pyrazole, see: Munakata *et al.* (1995 ▶); Nakano *et al.* (2004 ▶); Du *et al.* (2005 ▶); Yoneda, Adachi, Hayami *et al.* (2006 ▶); Yoneda, Adachi, Nishio *et al.* (2006 ▶); Ishikawa *et al.* (2008 ▶, 2010 ▶). For an example of a coordinated hydrogensulfate ion, see: Dragancea *et al.* (2008 ▶). 
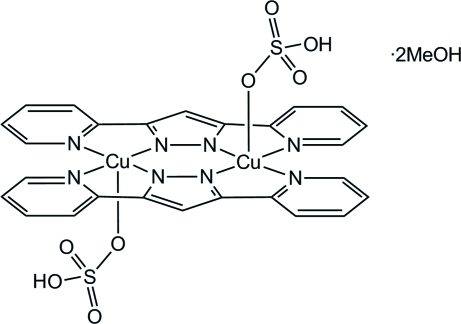


## Experimental

### 

#### Crystal data

[Cu_2_(C_13_H_9_N_4_)_2_(HO_4_S)_2_]·2CH_4_O
*M* *_r_* = 827.82
Monoclinic, 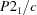

*a* = 6.0909 (3) Å
*b* = 16.0581 (6) Å
*c* = 15.6579 (7) Å
β = 95.2044 (14)°
*V* = 1525.16 (12) Å^3^
*Z* = 2
Mo *K*α radiation
μ = 1.61 mm^−1^
*T* = 200 K
0.35 × 0.04 × 0.03 mm

#### Data collection

Rigaku R-AXIS RAPID diffractometer
Absorption correction: multi-scan (*ABSCOR*; Rigaku, 1995 ▶) *T*_min_ = 0.926, *T*_max_ = 0.953
16136 measured reflections
2470 independent reflections
3262 reflections with *I* > 2σ(*I*)
*R*_int_ = 0.021

#### Refinement

*R*[*F* ^2^ > 2σ(*F* ^2^)] = 0.024
*wR*(*F* ^2^) = 0.068
*S* = 1.08
3470 reflections
227 parameters
H-atom parameters constrained
Δρ_max_ = 0.39 e Å^−3^
Δρ_min_ = −0.51 e Å^−3^

### 

Data collection: *RAPID-AUTO* (Rigaku, 2002 ▶); cell refinement: *RAPID-AUTO*; data reduction: *RAPID-AUTO*; program(s) used to solve structure: *SHELXS97* (Sheldrick, 2008 ▶); program(s) used to refine structure: *SHELXL97* (Sheldrick, 2008 ▶); molecular graphics: *CrystalStructure* (Rigaku, 2010 ▶); software used to prepare material for publication: *CrystalStructure*.

## Supplementary Material

Crystal structure: contains datablock(s) I, global. DOI: 10.1107/S1600536811038700/gk2397sup1.cif

Supplementary material file. DOI: 10.1107/S1600536811038700/gk2397Isup2.cdx

Structure factors: contains datablock(s) I. DOI: 10.1107/S1600536811038700/gk2397Isup2.hkl

Additional supplementary materials:  crystallographic information; 3D view; checkCIF report

## Figures and Tables

**Table 1:** Selected geometric parameters (Å, °)

Cu1—O1	2.2696 (13)
Cu1—N1	2.0865 (13)
Cu1—N2	1.9558 (13)
Cu1—N3^i^	1.9507 (13)
Cu1—N4^i^	2.0924 (14)

Symmetry code: (i) 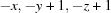.

**Fig. 1.**
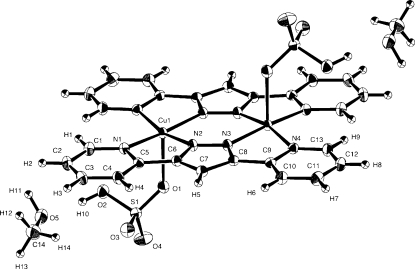
ORTEP drawing for the title complex showing 50% probability displacement ellipsoids. Symmentry code for unlabelled atoms: -x, -y+1, -z+1.

**Fig. 2.**
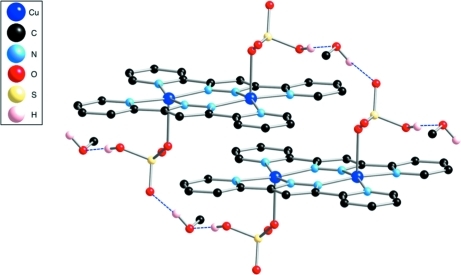
A fragment of one-dimensional chain structure of the title compound. Hydrogen bonds are shown as dashed lines.

**Fig. 3.**
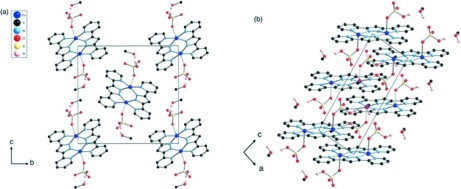
Crystal structure for the title compound viewed along the a axis (a) and the b axis (b). The C-bound hydrogen atoms have been omitted.

## supplementary crystallographic information

### Comment

3,5-Bis(2-pyridyl)pyrazole[Hbpypz] is a versatile ligand in the construction of a series of mononuclear, dinuclear and polynuclear complexes (Munakata *et al.*, 1995; Du *et al.*, 2005; Yoneda *et al.*, 2006; Ishikawa *et al.*, 2010). The dinuclear complexes show the structure where two bpypz^-^ ions are bridging two metal ions with the axial coordination sites. This kind of dinuclear complexes with transition metal ions were reported previously (Nakano *et al.*, 2004; Yoneda *et al.*, 2006; Ishikawa *et al.*, 2008). The title compound consists of the Cu^II^ dinuclear complex and two methanol solvent molecules. In the dinuclear complex, four N donors from two deprotonated tetradentate bridging bpypz^-^ ligands form the basal plane (Table 1). The copper(II) ion is penta-coordinated and it is in a slightly deformed square-pyramidal coordination environment with the τ value of 0.001 (Fig. 1). The apical position is occupied by the hydrogensulfate ion. An uncommon feature is that a sulfate ion is actually bound as the hydrogensulfate ion as there are only a few instances of unidentately coordinated hydrogensulfate ion (Dragancea *et al.*, 2008). The π···π stacking interactions between pyridyl and pyrazole rings [centroid-centroid distance 3.391 (3) Å ] and strong hydrogen bonds between the hydrogensulfate ligands and the methanol molecules assemble molecules into a one dimensional polymeric structure extended along the *a* axis (Table 2, Fig. 2). The methanol molecule acts both as an accepter and a donor in O-H···O hydrogen bond.

### Experimental

A methanolic solution of CuSO_4_.6H_2_O (5ml, 10 mmol*L*^-1^) was transferred to a glass tube, and then a methanolic solution of Hbpypz (5ml, 10 mmol*L*^-1^) was poured into the glass tube without mixing the solutions. Green crystals began to form at ambient temperature within one week (yield 84%). Elemental analysis: calcd (%) for C_28_H_28_Cu_2_N_8_O_10_S_2_: C 40.62, H 3.41, N 13.54; found: C 40.45, H 3.28, N 13.60.

### Refinement

The C-bound hydrogen atoms in the bpypz^-^ ion and the methyl group of the methanol molecule were placed at calculated positions, C—H 0.950 Å and 0.980 Å respectively, and were treated as riding on their parent atoms with *U*_iso_(H) set to 1.2 *U*_eq_(C). The O-bound hydrogen atoms in the hydrogensulfate ion and the methanol molecule were located in a difference Fourier map. The H-atom coordinates were fixed. The distances were O2–H10 0.90 Å and O5–H11 0.96 Å.

### Crystal data

**Table:**

[Cu_2_(C_13_H_9_N_4_)_2_(HO_4_S)_2_]·2CH_4_O	*F*(000) = 844.00
*M**_r_* = 827.82	*D*_x_ = 1.803 Mg m^−^^3^
Monoclinic, *P*2_1_/*c*	Mo *K*α radiation, λ = 0.71075 Å
Hall symbol: -P 2ybc	Cell parameters from 14158 reflections
*a* = 6.0909 (3) Å	θ = 3.4–27.5°
*b* = 16.0581 (6) Å	µ = 1.61 mm^−^^1^
*c* = 15.6579 (7) Å	*T* = 200 K
β = 95.2044 (14)°	Column, green
*V* = 1525.16 (12) Å^3^	0.35 × 0.04 × 0.03 mm
*Z* = 2	

### Data collection

**Table:**

Rigaku R-AXIS RAPID diffractometer	3262 reflections with *F*^2^ > 2.0σ(*F*^2^)
Detector resolution: 10.000 pixels mm^-1^	*R*_int_ = 0.021
ω scans	θ_max_ = 27.4°
Absorption correction: multi-scan (*ABSCOR*; Rigaku, 1995)	*h* = −7→7
*T*_min_ = 0.926, *T*_max_ = 0.953	*k* = −20→20
16136 measured reflections	*l* = −20→20
2470 independent reflections	

### Refinement

**Table:**

Refinement on *F*^2^	Secondary atom site location: difference Fourier map
*R*[*F*^2^ > 2σ(*F*^2^)] = 0.024	Hydrogen site location: inferred from neighbouring sites
*wR*(*F*^2^) = 0.068	H-atom parameters constrained
*S* = 1.08	*w* = 1/[σ^2^(*F*_o_^2^) + (0.0383*P*)^2^ + 0.881*P*] where *P* = (*F*_o_^2^ + 2*F*_c_^2^)/3
3470 reflections	(Δ/σ)_max_ = 0.001
227 parameters	Δρ_max_ = 0.39 e Å^−^^3^
0 restraints	Δρ_min_ = −0.51 e Å^−^^3^
Primary atom site location: structure-invariant direct methods	

### Special details

**Table:**

Geometry. ENTER SPECIAL DETAILS OF THE MOLECULAR GEOMETRY
Refinement. Refinement was performed using all reflections. The weighted *R*-factor(*wR*) and goodness of fit (*S*) are based on *F*^2^. *R*-factor (gt) are based on *F*. The threshold expression of *F*^2^ > 2.0 σ(*F*^2^) is used only for calculating *R*-factor (gt).

### Fractional atomic coordinates and isotropic or equivalent isotropic displacement parameters (Å^2^)

**Table:**

	*x*	*y*	*z*	*U*_iso_*/*U*_eq_	
Cu1	0.28031 (3)	0.520598 (11)	0.578370 (12)	0.01532 (7)	
S1	0.20037 (6)	0.55399 (2)	0.78731 (2)	0.01893 (10)	
O1	0.1195 (2)	0.56027 (8)	0.69710 (8)	0.0266 (3)	
O2	0.4401 (2)	0.58901 (8)	0.78864 (8)	0.0293 (3)	
O3	0.2141 (3)	0.46998 (8)	0.81989 (9)	0.0292 (3)	
O4	0.0797 (3)	0.60921 (9)	0.84007 (9)	0.0356 (4)	
O5	0.7222 (2)	0.55846 (10)	0.91528 (9)	0.0331 (3)	
N1	0.4667 (3)	0.42870 (8)	0.64544 (9)	0.0172 (3)	
N2	0.0927 (2)	0.42369 (8)	0.55066 (9)	0.0175 (3)	
N3	−0.1001 (2)	0.41178 (8)	0.50440 (9)	0.0172 (3)	
N4	−0.4729 (3)	0.37148 (8)	0.42106 (9)	0.0180 (3)	
C1	0.6627 (3)	0.43350 (10)	0.69186 (11)	0.0222 (4)	
C2	0.7564 (3)	0.36790 (11)	0.73994 (11)	0.0243 (4)	
C3	0.6436 (3)	0.29342 (11)	0.74028 (11)	0.0249 (4)	
C4	0.4411 (3)	0.28646 (10)	0.69234 (11)	0.0221 (4)	
C5	0.3577 (3)	0.35468 (10)	0.64594 (10)	0.0168 (3)	
C6	0.1491 (3)	0.35246 (9)	0.59261 (10)	0.0170 (3)	
C7	−0.0117 (3)	0.29197 (9)	0.57300 (10)	0.0186 (3)	
C8	−0.1656 (3)	0.33282 (9)	0.51668 (10)	0.0167 (3)	
C9	−0.3750 (3)	0.30969 (9)	0.47033 (10)	0.0169 (3)	
C10	−0.4679 (3)	0.23107 (10)	0.47581 (11)	0.0226 (4)	
C11	−0.6675 (3)	0.21423 (11)	0.42906 (12)	0.0262 (4)	
C12	−0.7683 (3)	0.27685 (11)	0.37925 (12)	0.0256 (4)	
C13	−0.6672 (3)	0.35401 (11)	0.37704 (11)	0.0232 (4)	
C14	0.7361 (4)	0.47382 (13)	0.94479 (15)	0.0361 (5)	
H1	0.7415	0.4846	0.6918	0.0267*	
H2	0.8955	0.3741	0.7720	0.0292*	
H3	0.7036	0.2475	0.7729	0.0299*	
H4	0.3610	0.2357	0.6914	0.0266*	
H5	−0.0154	0.2363	0.5932	0.0223*	
H6	−0.3957	0.1894	0.5111	0.0272*	
H7	−0.7334	0.1608	0.4313	0.0314*	
H8	−0.9054	0.2671	0.3469	0.0308*	
H9	−0.7386	0.3966	0.3428	0.0278*	
H10	0.5306	0.5733	0.8344	0.0351*	
H11	0.8529	0.5697	0.8873	0.0397*	
H12	0.8658	0.4471	0.9240	0.0434*	
H13	0.6029	0.4436	0.9229	0.0434*	
H14	0.7489	0.4730	1.0076	0.0434*	

### Atomic displacement parameters (Å^2^)

**Table:**

	*U*^11^	*U*^22^	*U*^33^	*U*^12^	*U*^13^	*U*^23^
Cu1	0.01457 (11)	0.01325 (11)	0.01755 (11)	−0.00117 (6)	−0.00178 (7)	0.00169 (6)
S1	0.02020 (19)	0.01756 (19)	0.01917 (19)	−0.00183 (14)	0.00250 (15)	−0.00195 (14)
O1	0.0213 (6)	0.0362 (7)	0.0219 (6)	0.0049 (6)	−0.0005 (5)	−0.0002 (5)
O2	0.0235 (6)	0.0335 (7)	0.0299 (7)	−0.0093 (6)	−0.0023 (5)	0.0060 (6)
O3	0.0320 (7)	0.0207 (6)	0.0344 (8)	−0.0032 (5)	0.0007 (6)	0.0053 (5)
O4	0.0447 (8)	0.0269 (7)	0.0380 (8)	−0.0005 (6)	0.0189 (7)	−0.0097 (6)
O5	0.0244 (7)	0.0382 (8)	0.0362 (8)	−0.0030 (6)	−0.0003 (6)	0.0022 (6)
N1	0.0175 (6)	0.0153 (6)	0.0187 (7)	0.0016 (5)	0.0003 (5)	−0.0006 (5)
N2	0.0169 (6)	0.0155 (6)	0.0195 (7)	−0.0008 (5)	−0.0028 (5)	0.0018 (5)
N3	0.0171 (6)	0.0146 (6)	0.0191 (7)	−0.0022 (5)	−0.0022 (5)	0.0017 (5)
N4	0.0166 (6)	0.0173 (6)	0.0202 (7)	−0.0014 (5)	0.0014 (5)	0.0007 (5)
C1	0.0189 (8)	0.0202 (8)	0.0269 (9)	0.0003 (6)	−0.0019 (7)	−0.0028 (7)
C2	0.0204 (8)	0.0268 (9)	0.0244 (9)	0.0040 (7)	−0.0052 (7)	−0.0028 (7)
C3	0.0255 (9)	0.0247 (9)	0.0233 (9)	0.0054 (7)	−0.0043 (7)	0.0042 (7)
C4	0.0236 (8)	0.0190 (8)	0.0232 (8)	0.0006 (7)	−0.0016 (7)	0.0035 (6)
C5	0.0182 (7)	0.0164 (7)	0.0161 (7)	0.0013 (6)	0.0022 (6)	−0.0003 (6)
C6	0.0182 (7)	0.0154 (7)	0.0174 (7)	0.0015 (6)	0.0012 (6)	0.0021 (6)
C7	0.0205 (8)	0.0149 (7)	0.0203 (8)	−0.0004 (6)	0.0009 (6)	0.0022 (6)
C8	0.0183 (7)	0.0141 (7)	0.0178 (7)	−0.0015 (6)	0.0026 (6)	0.0006 (6)
C9	0.0175 (7)	0.0160 (7)	0.0173 (7)	−0.0006 (6)	0.0030 (6)	−0.0011 (6)
C10	0.0238 (8)	0.0172 (7)	0.0267 (9)	−0.0021 (7)	0.0010 (7)	0.0006 (6)
C11	0.0254 (9)	0.0208 (8)	0.0322 (10)	−0.0082 (7)	0.0020 (7)	−0.0031 (7)
C12	0.0186 (8)	0.0290 (9)	0.0286 (9)	−0.0070 (7)	−0.0020 (7)	−0.0023 (7)
C13	0.0185 (8)	0.0248 (8)	0.0254 (8)	−0.0021 (7)	−0.0027 (7)	0.0026 (7)
C14	0.0367 (11)	0.0384 (12)	0.0331 (11)	−0.0026 (9)	0.0014 (8)	0.0057 (9)

### Geometric parameters (Å, °)

**Table:**

Cu1—O1	2.2696 (13)	C6—C7	1.394 (2)
Cu1—N1	2.0865 (13)	C7—C8	1.392 (2)
Cu1—N2	1.9558 (13)	C8—C9	1.457 (2)
Cu1—N3^i^	1.9507 (13)	C9—C10	1.389 (3)
Cu1—N4^i^	2.0924 (14)	C10—C11	1.387 (3)
S1—O1	1.4566 (13)	C11—C12	1.382 (3)
S1—O2	1.5630 (13)	C12—C13	1.385 (3)
S1—O3	1.4420 (14)	O2—H10	0.900
S1—O4	1.4557 (16)	O5—H11	0.960
O5—C14	1.436 (3)	C1—H1	0.950
N1—C1	1.342 (2)	C2—H2	0.950
N1—C5	1.362 (2)	C3—H3	0.950
N2—N3	1.3368 (18)	C4—H4	0.950
N2—C6	1.348 (2)	C7—H5	0.950
N3—C8	1.348 (2)	C10—H6	0.950
N4—C9	1.361 (2)	C11—H7	0.950
N4—C13	1.344 (2)	C12—H8	0.950
C1—C2	1.387 (3)	C13—H9	0.950
C2—C3	1.380 (3)	C14—H12	0.980
C3—C4	1.389 (3)	C14—H13	0.980
C4—C5	1.385 (3)	C14—H14	0.980
C5—C6	1.457 (2)		
			
O1—Cu1—N1	92.40 (5)	N2—C6—C7	109.97 (13)
O1—Cu1—N2	96.79 (6)	C5—C6—C7	134.63 (14)
O1—Cu1—N3^i^	97.44 (6)	C6—C7—C8	103.33 (13)
O1—Cu1—N4^i^	92.72 (5)	N3—C8—C7	110.04 (13)
N1—Cu1—N2	80.16 (6)	N3—C8—C9	115.19 (13)
N1—Cu1—N3^i^	167.37 (6)	C7—C8—C9	134.77 (14)
N1—Cu1—N4^i^	107.72 (6)	N4—C9—C8	114.53 (13)
N2—Cu1—N3^i^	90.77 (6)	N4—C9—C10	122.53 (14)
N2—Cu1—N4^i^	167.42 (6)	C8—C9—C10	122.94 (14)
N3^i^—Cu1—N4^i^	79.82 (6)	C9—C10—C11	119.12 (15)
O1—S1—O2	102.78 (7)	C10—C11—C12	118.63 (16)
O1—S1—O3	114.30 (8)	C11—C12—C13	119.29 (16)
O1—S1—O4	111.38 (8)	N4—C13—C12	123.13 (16)
O2—S1—O3	107.92 (8)	N1—C1—H1	118.240
O2—S1—O4	107.00 (8)	C2—C1—H1	118.243
O3—S1—O4	112.66 (9)	C1—C2—H2	120.658
Cu1—O1—S1	129.93 (8)	C3—C2—H2	120.663
Cu1—N1—C1	130.43 (11)	C2—C3—H3	120.467
Cu1—N1—C5	112.14 (10)	C4—C3—H3	120.467
C1—N1—C5	117.20 (14)	C3—C4—H4	120.496
Cu1—N2—N3	134.51 (11)	C5—C4—H4	120.484
Cu1—N2—C6	116.71 (10)	C6—C7—H5	128.336
N3—N2—C6	108.36 (13)	C8—C7—H5	128.337
Cu1^i^—N3—N2	133.96 (11)	C9—C10—H6	120.442
Cu1^i^—N3—C8	117.54 (10)	C11—C10—H6	120.440
N2—N3—C8	108.31 (13)	C10—C11—H7	120.683
Cu1^i^—N4—C9	112.64 (10)	C12—C11—H7	120.692
Cu1^i^—N4—C13	129.97 (12)	C11—C12—H8	120.351
C9—N4—C13	117.30 (14)	C13—C12—H8	120.364
N1—C1—C2	123.52 (15)	N4—C13—H9	118.439
C1—C2—C3	118.68 (16)	C12—C13—H9	118.430
C2—C3—C4	119.07 (16)	O5—C14—H12	109.477
C3—C4—C5	119.02 (15)	O5—C14—H13	109.477
N1—C5—C4	122.52 (14)	O5—C14—H14	109.472
N1—C5—C6	114.75 (14)	H12—C14—H13	109.472
C4—C5—C6	122.73 (15)	H12—C14—H14	109.469
N2—C6—C5	115.40 (13)	H13—C14—H14	109.460
			
O1—Cu1—N1—C1	−85.61 (12)	Cu1—N2—C6—C5	−6.86 (18)
O1—Cu1—N1—C5	88.62 (9)	Cu1—N2—C6—C7	173.75 (9)
N1—Cu1—O1—S1	26.68 (10)	N3—N2—C6—C5	179.56 (12)
O1—Cu1—N2—N3	88.20 (13)	N3—N2—C6—C7	0.17 (17)
O1—Cu1—N2—C6	−83.23 (10)	C6—N2—N3—Cu1^i^	−174.97 (13)
N2—Cu1—O1—S1	107.06 (10)	C6—N2—N3—C8	−0.31 (17)
O1—Cu1—N3^i^—N2^i^	−87.66 (13)	Cu1^i^—N3—C8—C7	176.01 (9)
O1—Cu1—N3^i^—C8^i^	86.62 (10)	Cu1^i^—N3—C8—C9	−4.37 (18)
N3^i^—Cu1—O1—S1	−161.27 (10)	N2—N3—C8—C7	0.34 (17)
O1—Cu1—N4^i^—C9^i^	−92.61 (9)	N2—N3—C8—C9	179.96 (12)
O1—Cu1—N4^i^—C13^i^	83.70 (12)	Cu1^i^—N4—C9—C8	3.47 (16)
N4^i^—Cu1—O1—S1	−81.19 (10)	Cu1^i^—N4—C9—C10	−176.54 (10)
N1—Cu1—N2—N3	179.47 (14)	Cu1^i^—N4—C13—C12	175.54 (10)
N1—Cu1—N2—C6	8.03 (9)	C9—N4—C13—C12	−0.6 (3)
N2—Cu1—N1—C1	177.90 (13)	C13—N4—C9—C8	−179.71 (13)
N2—Cu1—N1—C5	−7.86 (9)	C13—N4—C9—C10	0.3 (3)
N1—Cu1—N4^i^—C9^i^	174.00 (8)	N1—C1—C2—C3	0.2 (3)
N1—Cu1—N4^i^—C13^i^	−9.69 (14)	C1—C2—C3—C4	0.3 (3)
N4^i^—Cu1—N1—C1	8.01 (14)	C2—C3—C4—C5	−0.3 (3)
N4^i^—Cu1—N1—C5	−177.75 (8)	C3—C4—C5—N1	−0.0 (3)
N2—Cu1—N3^i^—N2^i^	9.29 (14)	C3—C4—C5—C6	178.73 (14)
N2—Cu1—N3^i^—C8^i^	−176.42 (10)	N1—C5—C6—N2	−0.2 (2)
N3^i^—Cu1—N2—N3	−9.38 (14)	N1—C5—C6—C7	178.96 (15)
N3^i^—Cu1—N2—C6	179.19 (10)	C4—C5—C6—N2	−179.10 (14)
N3^i^—Cu1—N4^i^—C9^i^	4.46 (9)	C4—C5—C6—C7	0.1 (3)
N3^i^—Cu1—N4^i^—C13^i^	−179.23 (13)	N2—C6—C7—C8	0.03 (17)
N4^i^—Cu1—N3^i^—N2^i^	−179.10 (14)	C5—C6—C7—C8	−179.20 (17)
N4^i^—Cu1—N3^i^—C8^i^	−4.82 (9)	C6—C7—C8—N3	−0.22 (17)
O2—S1—O1—Cu1	47.83 (11)	C6—C7—C8—C9	−179.74 (16)
O3—S1—O1—Cu1	−68.82 (12)	N3—C8—C9—N4	0.3 (2)
O4—S1—O1—Cu1	162.08 (10)	N3—C8—C9—C10	−179.68 (13)
Cu1—N1—C1—C2	173.45 (10)	C7—C8—C9—N4	179.81 (17)
Cu1—N1—C5—C4	−174.59 (10)	C7—C8—C9—C10	−0.2 (3)
Cu1—N1—C5—C6	6.54 (16)	N4—C9—C10—C11	0.4 (3)
C1—N1—C5—C4	0.5 (3)	C8—C9—C10—C11	−179.63 (14)
C1—N1—C5—C6	−178.39 (13)	C9—C10—C11—C12	−0.7 (3)
C5—N1—C1—C2	−0.5 (3)	C10—C11—C12—C13	0.4 (3)
Cu1—N2—N3—Cu1^i^	13.1 (3)	C11—C12—C13—N4	0.3 (3)
Cu1—N2—N3—C8	−172.25 (11)		

Symmetry codes: (i) −*x*, −*y*+1, −*z*+1.

### Hydrogen-bond geometry (Å, °)

**Table:**

*D*—H···*A*	*D*—H	H···*A*	*D*···*A*	*D*—H···*A*
O2—H10···O5	0.90	1.66	2.5509 (18)	170
O5—H11···O4^ii^	0.96	1.74	2.694 (2)	169

Symmetry codes: (ii) *x*+1, *y*, *z*.
